# Deregulation of *S*-adenosylmethionine biosynthesis and regeneration improves methylation in the *E. coli* de novo vanillin biosynthesis pathway

**DOI:** 10.1186/s12934-016-0459-x

**Published:** 2016-04-11

**Authors:** Aditya M. Kunjapur, Jason C. Hyun, Kristala L. J. Prather

**Affiliations:** Department of Chemical Engineering, Massachusetts Institute of Technology, 77 Massachusetts Avenue, Room E17-504G, Cambridge, MA 02139 USA; Synthetic Biology Engineering Research Center (SynBERC), Massachusetts Institute of Technology, Cambridge, MA 02139 USA; Department of Genetics, Harvard Medical School, Boston, MA 02115 USA

**Keywords:** *E. coli*, Vanillin, Methylation, *S*-adenosylmethionine, SAM, AdoMet, Methionine, Deregulation, Metabolic engineering

## Abstract

**Background:**

Vanillin is an industrially valuable molecule that can be produced from simple carbon sources in engineered microorganisms such as *Saccharomyces cerevisiae* and *Escherichia coli*. In *E. coli*, de novo production of vanillin was demonstrated previously as a proof of concept. In this study, a series of data-driven experiments were performed in order to better understand limitations associated with biosynthesis of vanillate, which is the immediate precursor to vanillin.

**Results:**

Time-course experiments monitoring production of heterologous metabolites in the *E. coli* de novo vanillin pathway revealed a bottleneck in conversion of protocatechuate to vanillate. Perturbations in central metabolism intended to increase flux into the heterologous pathway increased average vanillate titers from 132 to 205 mg/L, but protocatechuate remained the dominant heterologous product on a molar basis. SDS-PAGE, in vitro activity measurements, and l-methionine supplementation experiments suggested that the decline in conversion rate was influenced more by limited availability of the co-substrate *S*-adenosyl-l-methionine (AdoMet or SAM) than by loss of activity of the heterologous *O*-methyltransferase. The combination of *metJ* deletion and overexpression of feedback-resistant variants of *metA* and *cysE*, which encode enzymes involved in SAM biosynthesis, increased average de novo vanillate titers by an additional 33 % (from 205 to 272 mg/L). An orthogonal strategy intended to improve SAM regeneration through overexpression of native *mtn* and *luxS* genes resulted in a 25 % increase in average de novo vanillate titers (from 205 to 256 mg/L). Vanillate production improved further upon supplementation with methionine (as high as 419 ± 58 mg/L), suggesting potential for additional enhancement by increasing SAM availability.

**Conclusions:**

Results from this study demonstrate context dependency of engineered pathways and highlight the limited methylation capacity of *E. coli*. Unlike in previous efforts to improve SAM or methionine biosynthesis, we pursued two orthogonal strategies that are each aimed at deregulating multiple reactions. Our results increase the working knowledge of SAM biosynthesis engineering and provide a framework for improving titers of metabolic products dependent upon methylation reactions.

**Electronic supplementary material:**

The online version of this article (doi:10.1186/s12934-016-0459-x) contains supplementary material, which is available to authorized users.

## Background

Few biological molecules are as widely savored by humans as vanillin. Vanillin is the dominant flavor constituent in natural vanilla extract and the most heavily used food additive for flavoring annually by volume [1, 2]. Vanillin also has a variety of industrial uses, including as an intermediate in the chemical and pharmaceutical industries for production of herbicides, antifoaming agents, and drugs [3]. Biosynthesis of vanillin occurs naturally in the tropical vanilla orchid (*Vanilla planifolia*) and in trace amounts in other plants [3]. In *V. planifolia*, vanillin forms inside inedible pods or beans from which it must be extracted. Vanilla beans are harvested roughly 6–8 months after pollination and are subject to a curing process that can take more than 6 months to complete [3]. Inefficiencies stemming from *V. planifolia* cultivation are manifested in the high price of natural vanilla extract, which is estimated to be between $1000 and $4000 per kilogram [3]. Given that, researchers have sought to develop biosynthetic routes to vanillin in other host organisms. Ideally, these heterologous hosts could produce vanillin from glucose as a sole carbon source (de novo biosynthesis) given the abundance and affordability of glucose relative to other potential substrates [4].

De novo vanillin biosynthesis has been demonstrated in two of the industrial biotechnology workhorse organisms: *Saccharomyces cerevisiae* (baker’s yeast) [4] and *Escherichia coli* [5]. De novo production of vanillin in a heterologous host organism was first reported in both *S. cerevisiae* and *Schizosaccharomyces pombe* in 2009 [4]. The engineered metabolic pathway in yeast featured three heterologous enzymes: a dehydroshikimate dehydratase, an *O*-methyltransferase, and a carboxylic acid reductase. This pathway is similar to the *E. coli* pathway engineered by Frost and co-workers in 1998 [6]. The *E. coli* pathway was designed to produce vanillate in vivo using a heterologous dehydroshikimate dehydratase and *O*-methyltransferase. Subsequent reduction of purified vanillate into vanillin was catalyzed by a carboxylic acid reductase in vitro. The presumed rationale for the original two-stage vanillin pathway was that *E. coli* exhibits a high degree of aldehyde reductase activity catalyzed by numerous unknown endogenous enzymes, which would result in formation of vanillyl alcohol in vivo instead of vanillin. The problem of endogenous aromatic aldehyde reduction was recently addressed [5, 7], leading to a strain capable of accumulating aldehydes including vanillin (*E. coli* RARE, Addgene Catalog #61440). At that time, the de novo vanillin pathway was constructed in *E. coli* as one of two demonstrated and industrially relevant applications for the RARE strain.

When de novo biosynthesis was first reported in yeast and in *E. coli*, vanillin titers achieved were similarly low under comparable flask-scale conditions (65 mg/L in *S. pombe* supplied with yeast extract-based media [4] versus 119 or 56 mg/L in *E. coli* supplied with glucose in LB or M9 minimal media [5], respectively). Production of vanillin or vanillin-β-d-glucoside in yeast has since been enhanced [8, 9] and even commercialized by the company Evolva [10]. Numerous differences between eukaryotic yeast and prokaryotic *E. coli* led us to suspect that similar de novo pathways might suffer from different host-dependent limitations. Metabolic engineering reviews that discuss host selection not only compare natural differences in metabolic fluxes and regulation across model organisms such as yeast and *E. coli* [11] but also highlight dissimilarities in these respects among different species of yeast [12] and even among different strains of *E. coli* [13].

**Fig. 1:**
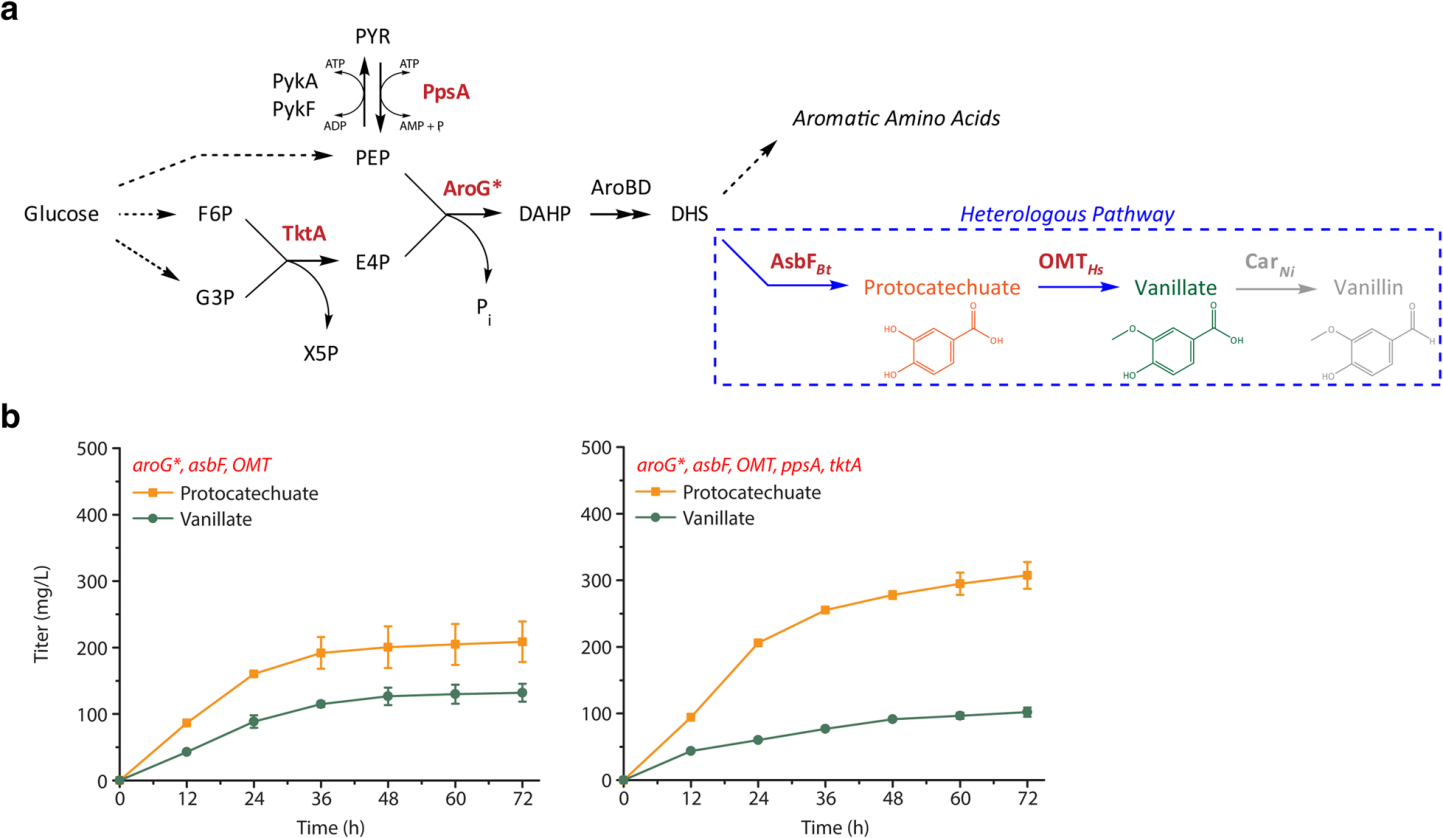
Conversion of protocatechuate to vanillate limits engineered de novo vanillin biosynthesis in *E. coli*. **a** Metabolic pathway diagram depicting engineered route from glucose to vanillin. Genes corresponding to enzymes labeled in *red* are overexpressed in this study. Enzymes written without subscripts are native to *E. coli*. *Dashed blue lines* indicate the heterologous portion of the pathway. The gene corresponding to Car_*Ni*_ (shown in *gray*) is not overexpressed throughout this study to avoid confounding presence of aldehydes during pathway troubleshooting. Thus, only *solid blue arrows* represent heterologous reaction steps pertaining to this study, where vanillate is the desired end product. Maximum theoretical yields of vanillin and vanillate using this pathway are 0.395 and 0.414 mol/mol_glucose, respectively. **b** Time-course experiment in which the PTS^−^ glu^+^ RAREʹ host strain was transformed to express aroG*, *asbF*_*Bt*_, and *OMT*_*Hs*_ or *ppsA* and *tktA* in addition to the other genes. Titers of heterologous metabolites protocatechuate and vanillate were measured every 12 h by HPLC and reveal bottleneck in conversion of protocatechuate to vanillate. Genes overexpressed in each experiment are labeled in *red*. Titers of protocatechuate increase by roughly 50 % when *ppsA* and *tktA* are overexpressed, suggesting flux into the heterologous pathway increased. However, vanillate titers do not improve, motivating subsequent focus on conversion of protocatechuate to vanillate

In this report, we describe a series of metabolic engineering experiments used to improve the production of vanillate from glucose as a sole carbon source using *E. coli*. We initially focus on increasing flux into the engineered pathway by perturbing pool sizes of precursor molecules and observing the effect on heterologous metabolite titers as a function of time in order to identify pathway bottlenecks downstream. To simplify our analysis, we exclude the confounding presence of aldehyde products, which exert negative effects on titer by inhibiting cell growth [5, 14]. We achieve this by omitting the carboxylic acid reductase Car_*Ni*_ from the pathway, which results in vanillate as the intended final product rather than vanillin (Fig. 1a).

Our findings indicate that limited availability of *S*-adenosylmethionine (SAM) represents a major hurdle to the improvement of vanillate titers from glucose as a sole carbon source in *E. coli*. To improve vanillate titers, we deregulate reactions in SAM biosynthesis and regeneration using two orthogonal strategies. Improvements in vanillate formation as a function of increased SAM availability have impact beyond the vanillin pathway given that SAM-dependent methyltransferases constitute a broad class of enzymes with potential for engineering numerous metabolic products [15, 16]. Our results increase the working knowledge of SAM biosynthesis engineering and provide a framework for improving titers of metabolic products dependent upon methylation reactions.

## Results

### Time-course experiments monitoring heterologous metabolites reveal limitation in conversion of protocatechuate to vanillate

As a starting point for understanding pathway limitations, we sought to increase carbon flux entering the heterologous portion of the vanillate pathway (Fig. 1a). We hypothesized that increasing flux would facilitate the identification of pathway bottlenecks based on changes in titer (units of mg/L) and specific yields [units of g/g_DCW_ (grams of dry cell weight)] of intermediate metabolites. In our previous demonstration of de novo vanillin biosynthesis, titers of all measured heterologous metabolites (protocatechuate, vanillate, protocatechualdehyde, and vanillin) were low, each less than 60 mg/L [5]. Initially, we sought to achieve increased heterologous metabolite titers by targeting endogenous precursor biosynthesis genes for overexpression given that the first heterologous enzyme (AsbF_*Bt*_) is reported to efficiently catalyze conversion of endogenous 3-dehydroshikimate into protocatechuate [17].

The heterologous vanillate pathway branches from endogenous aromatic amino acid biosynthesis, and efforts to improve aromatic amino acid biosynthesis in *E. coli* have been well-documented [18–20]. In our initial demonstration of vanillin synthesis, we harnessed one of these strategies by expressing a feedback-resistant variant of the enzyme that catalyzes the first committed step to aromatic amino acid biosynthesis (aroG*) [5]. Previous studies demonstrated two other strategies that were successfully used to increase titers of aromatic products, in these cases by increasing availability of two key aromatic precursor metabolites: phosphoenolpyruvate (PEP) from glycolysis and erythrose-4-phosphate (E4P) from the pentose phosphate pathway. PEP and E4P condense to form DAHP during the first committed step towards aromatic amino acid biosynthesis (Fig. 1a) [18–20]. One reported strategy to increase their availability is deletion of the phosphotransferase system (PTS), which is the primary means for glucose import and consumes 1 mole of PEP per mole of glucose. Growth of a PTS^−^ strain on glucose as a sole carbon source can be made viable by upregulating the gene encoding galactose permease (*galP*), which allows glucose entry independent of PEP consumption (PTS^−^ glu^+^). A second documented strategy to increase availability of PEP and E4P is to overexpress the genes encoding PEP synthase (*ppsA*) and transketolase (*tktA*) [18–20]. PEP synthase catalyzes the conversion of PEP into pyruvate, and transketolase catalyzes the reversible formation of E4P and xylulose 5-phosphate from fructose 6-phosphate and glyceraldehyde 3-phosphate.

To test these strategies, we engineered a PTS^−^ glu^+^ variant of the RARE strain (PTS^−^ glu^+^ RAREʹ), where the “RARE prime” designation indicates that this strain contains intact versions of two genes (*dkgB* and *yeaE*) that were targeted for deletion in the original RARE strain. Previous work found that these two genes did not substantially contribute to aldehyde reductase activity given their low native expression [5]. When the PTS^−^ glu^+^ RAREʹ host overexpressed the aroG*, *asbF*_*Bt*_, and *OMT*_*Hs*_ genes, 132 ± 14 mg/L of vanillate was produced 72 h after induction (Fig. 1b). When the same host was used to express the *ppsA* and *tktA* genes in addition to the previously mentioned pathway genes, vanillate titers slightly decreased but cumulative titers of heterologous metabolites increased. Specifically, protocatechuate titer increased from 200 to 300 mg/L in strains expressing the *ppsA* and *tktA* genes. In addition, the specific yield of protocatechuate noticeably increased around 24 h after pathway induction when *ppsA* and *tktA* were overexpressed (Additional file 1: Figure S1). In both experiments, protocatechuate was the dominant heterologous metabolite formed on a molar basis, and this suggested that the reaction catalyzed by the *O*-methyltransferase was limiting. It is possible that the decrease in vanillate titers resulted either from decreased *OMT*_*Hs*_ expression as two additional genes were being expressed, or from the decreased rate of biomass formation during expression of the two additional genes (Additional file 1: Figure S1), or from both contributions.

**Fig. 2:**
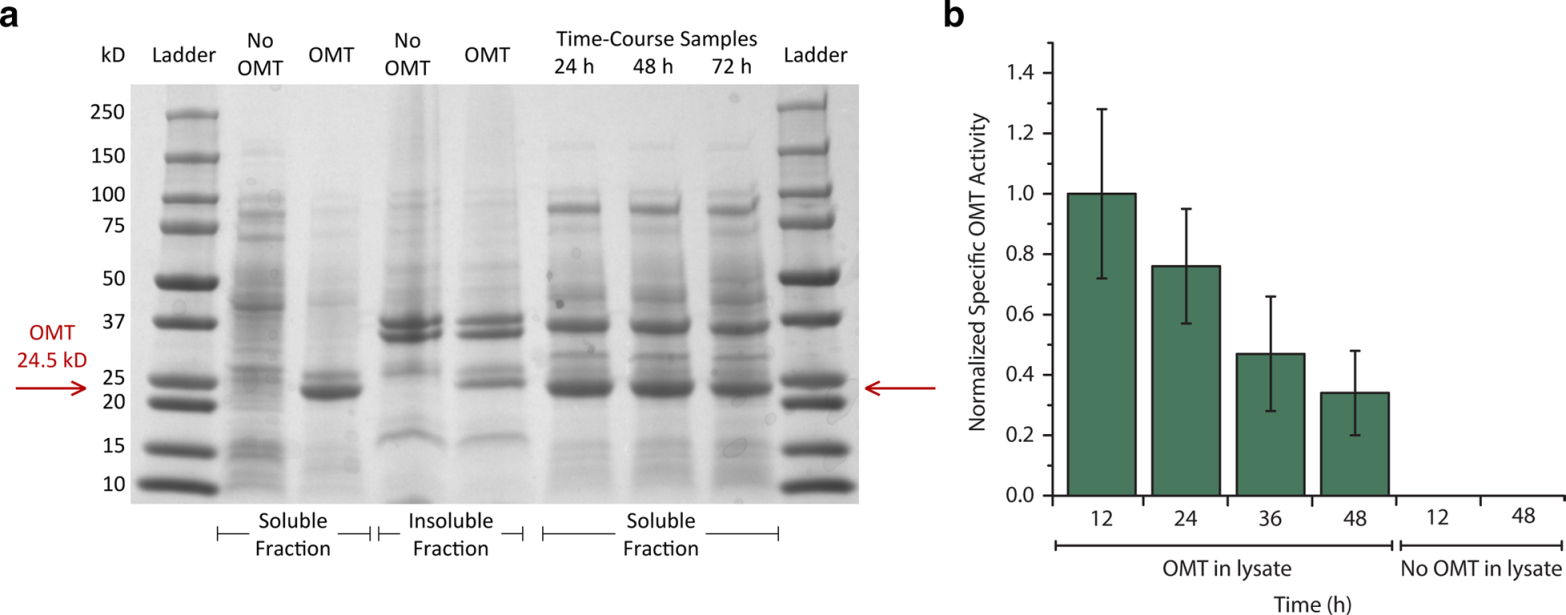
Expression and activity data suggests principal limitation is not heterologous *O*-methyltransferase. **a** SDS-PAGE gel suggesting robust OMT_*Hs*_ expression in soluble fractions of cells sampled from flask cultures. **b** In vitro specific activity measurements normalized to activity at 12 h time point. Activity data indicates that OMT_*Hs*_ activity declines from 12 to 48 h. However, notable activity even at 48 h strongly suggests that loss of OMT_*Hs*_ activity is not responsible for observed reduction in rate of vanillate formation

Although the gene encoding OMT_*Hs*_ was codon-optimized for expression in *E. coli*, we next wondered whether OMT_*Hs*_ may be expressing poorly or whether it may have low activity in *E. coli*. SDS-PAGE results suggested that OMT_*Hs*_ expresses well (Fig. 2a). In addition, cultures expressing the pathway were sampled for in vitro specific OMT_*Hs*_ activity. Activity measurements revealed that, while OMT_*Hs*_ activity decreases to 34 % of initially measured activity, the enzyme is still active 48 h after induction (Fig. 2b).

### Supplementation of methionine and homocysteine improve vanillate titers, strongly suggesting that availability of the co-substrate *S*-adenosylmethionine (SAM) limits conversion of protocatechuate to vanillate

**Fig. 3:**
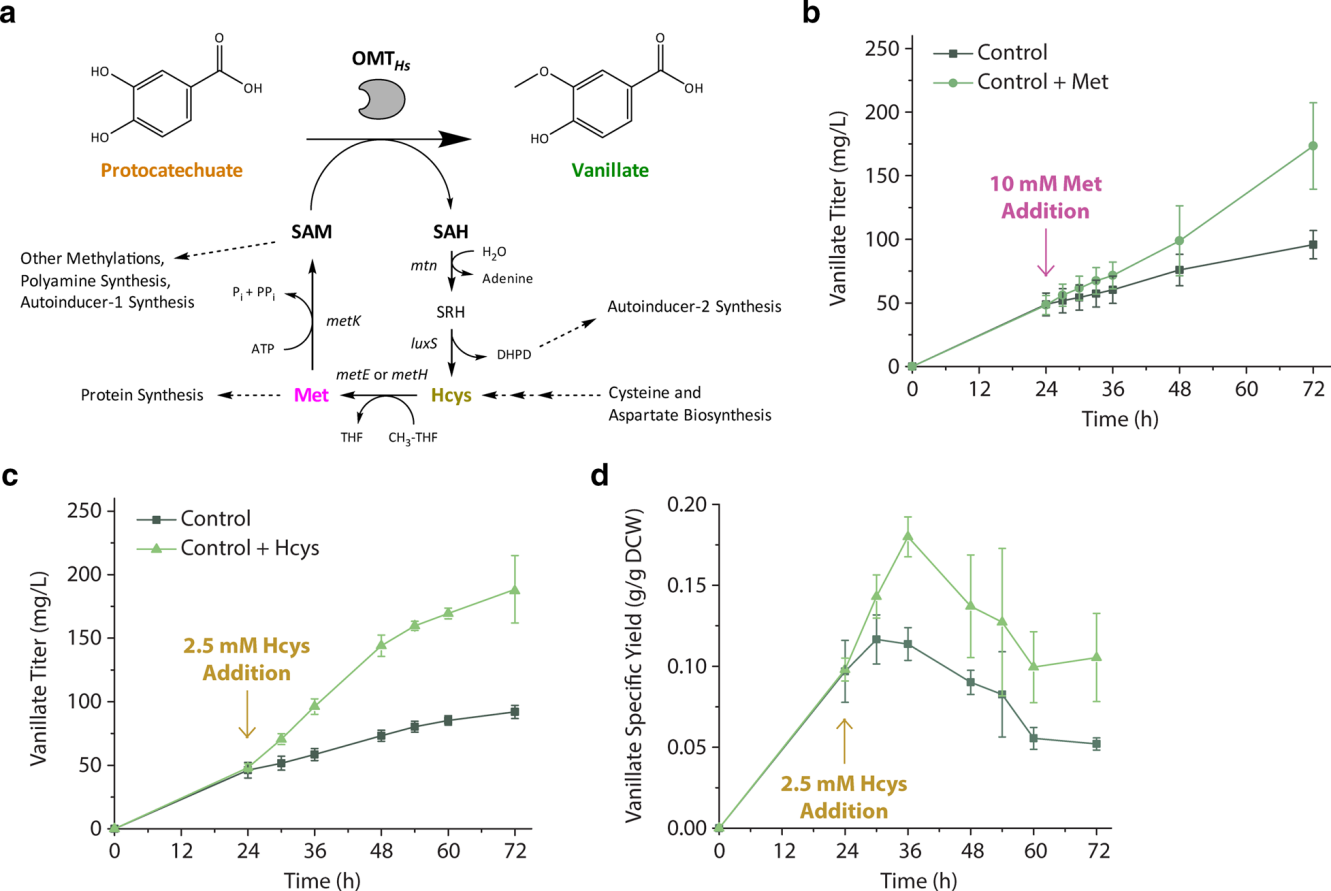
Supplementation experiments indicate limiting *S*-adenosylmethionine (SAM) availability and demonstrate SAM biosynthesis bottleneck upstream of homocysteine. **a** Metabolic pathway diagram illustrating co-substrate requirement for reaction catalyzed by *O*-methyltransferase. SAM is generated from methionine, which in turn is generated from homocysteine. **b** Vanillate titers resulting from the presence or absence of 10 mM methionine supplementation to cultures 24 h after induction. Cultures receiving methionine produced nearly twofold higher vanillate titers. **c** Vanillate titers resulting from the presence or absence of 2.5 mM homocysteine supplementation to cultures 24 h after induction. Lower concentrations of homocysteine were used relative to methionine given the potential for homocysteine toxicity. Once again, cultures receiving supplement produced nearly twofold higher vanillate titers. **d** Vanillate specific yields resulting from homocysteine supplementation experiment. Higher specific yield upon homocysteine supplementation demonstrates that increased vanillate production is due to greater output per cell and not because of additional biomass. These pathway experiments used the PTS^−^ glu^+^ RAREʹ host and overexpression of aroG*,*asbF*_*Bt*_, *OMT*_*Hs*_ *, ppsA,* and *tktA*

Given that OMT_*Hs*_ appeared to be expressed and active in cells at times when conversion of protocatechuate to vanillate was not occurring, we next considered that co-substrate availability may be limiting (Fig. 3a). The co-substrate SAM fulfills a variety of cellular roles, such as serving as the primary methyl donor for all cellular methylations, which compete directly with the engineered vanillin pathway. Methionine is endogenously converted to SAM by methionine adenosyltransferase (otherwise known as SAM synthetase) encoded by *metK*. Although exogenously supplied SAM does not enter *E. coli*, exogenously supplied methionine does enter cells and is known to indirectly perturb intracellular SAM availability in *E. coli* [6] and in yeast [21]. Importantly, in the sole previously reported effort to produce vanillate using *E. coli*, it was observed that methionine supplementation improved titers [6].

We monitored vanillate titers in cultures with and without supplementation of 10 mM l-methionine at peak vanillate productivity (24 h after induction) (Fig. 3b). Although methionine supplementation did not result in significant changes in vanillate titer immediately, final vanillate titer increased twofold 72 h after induction. In addition, vanillate synthesis occurred at a relatively constant rate until the final sampling time of 72 h upon methionine supplementation. This result suggests that potential depletion of SAM and methionine pools later in the culture may be responsible for limiting conversion of protocatechuate to vanillate.

To better understand the contributions of methionine biosynthesis to the vanillate pathway, a second supplementation experiment was performed, this time with the direct precursor to methionine, l-homocysteine (Fig. 3a). The methylation of homocysteine to form methionine is reported to be problematic under oxidative conditions due to the inactivation of catalytic residues in the cobalamin-independent methionine synthase (MetE) [22–25]. As before, cultures at peak vanillate productivity (24 h) were supplemented with and without l-homocysteine. Because l-homocysteine is reported to be toxic for *E. coli* [26, 27], we added 2.5 mM rather than 10 mM. Once again, supplemented cultures displayed an increase in vanillate titer (up to 200 mg/L) and an increase in duration of vanillate production consistent with what was observed for l-methionine addition (Fig. 3c). The average rate of vanillate synthesis doubled from 1.3 to 2.6 mg/L h. Importantly, vanillate specific yields showed that homocysteine supplementation led to higher specific production of vanillate rather than higher cell density (Fig. 3d). These results collectively suggested that reactions upstream of homocysteine synthesis in the methionine biosynthesis pathway need to be targeted to achieve increases in vanillate production from glucose as a sole carbon source.

**Fig. 4:**
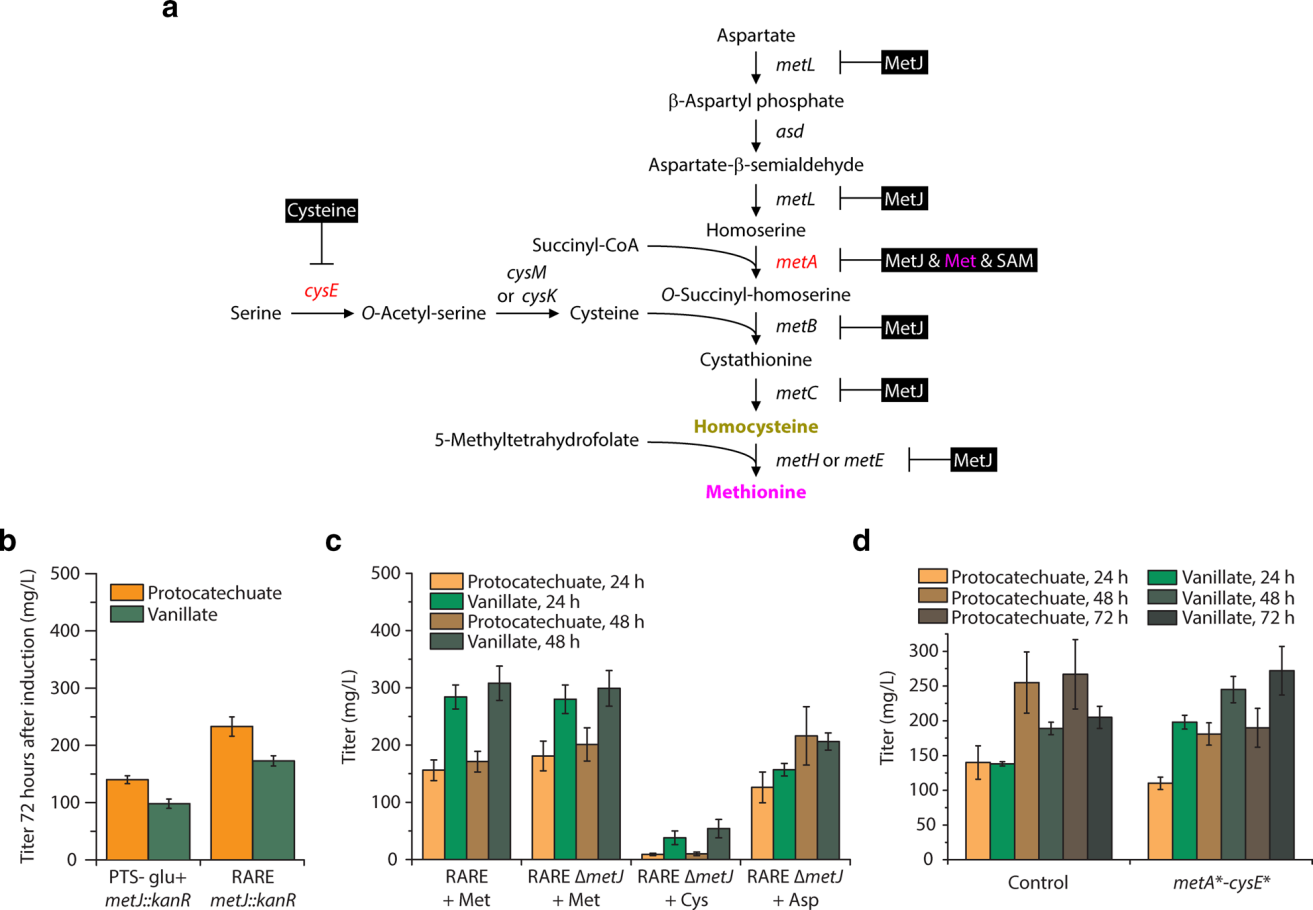
Effect of *metJ* deletion on protocatechuate and vanillate titers (**a**) in different host strains and (**b**) in the presence of amino acid supplementation. For these experiments, the following genes were overexpressed: aroG*,*asbF*_*Bt*_, *OMT*_*Hs*_ *, ppsA,* and *tktA*. For the amino acid supplementation experiment (**c**), 10 mM of amino acid was added at induction. **d** Effect of overexpressing feedback-desensitized variants of *metA* and *cysE* along with aroG*,*asbF*_*Bt*_, *OMT*_*Hs*_ *, ppsA,* and *tktA* in the RARE ∆*metJ* host. The control represents co-transformation with an empty pCOLADuet-1 plasmid

Unlike aromatic amino acid biosynthesis, methionine biosynthesis in *E. coli* and other bacteria is not well understood and is regulated at multiple transcriptional as well as post-transcriptional levels (Fig. 4a). In fact, until recently, methionine was the only essential amino acid that was not commercially produced using fermentative processes [28, 29]. The protein encoded by *metJ* is the primary transcriptional regulator of several genes involved in methionine biosynthesis [30–32]. Methionine titers of 910 mg/L were previously achieved using an *E. coli* strain that was constructed by mutagenesis with nitrosoguanidine along with selection based on resistance to methionine analogs. This strain was observed to have a mutation in *metJ* that decreased repression of methionine biosynthesis [33]. However, genome sequencing was not performed and it is unlikely that *metJ* was the only gene containing a mutation. In addition to regulation by *metJ*, MetA (homoserine succinyltransferase) catalyzes the first committed step in methionine biosynthesis and is reported to be inhibited by both methionine and SAM [34]. l-cysteine is also a precursor to methionine. CysE (l-serine *O*-acetyltransferase) catalyzes the first step of cysteine biosynthesis and is reported to display significant inhibition by cysteine [35]. Fortunately, variants of MetA and CysE that have been engineered to display desensitization to feedback inhibition (MetA* and CysE*) have been reported [34, 35]. The combination of metA* expression, *metJ* deletion, and other modifications have previously led to a strain that accumulated 240 mg/L of methionine [34].

### Deregulation of SAM biosynthesis through deletion of *metJ* and expression of metA* and cysE* improves vanillate titers

We hypothesized that vanillate titers might increase if methionine biosynthesis were modified by (i) deleting *metJ*; and (ii) expressing metA* and cysE* (Fig. 4a). Given our observation of slower growth in the PTS^−^ glu^+^ RAREʹ strain relative to the RARE strain, we decided to delete *metJ* in both strains and compare performance. Upon overexpression of aroG*, *asbF*, *OMT*, *ppsA*, and *tktA*, the RARE ∆*metJ* host resulted in higher titers of protocatechuate and vanillate than the PTS^−^ glu^+^ RAREʹ ∆*metJ* host (Fig. 4b). However, protocatechuate titers remained higher than vanillate titers in both cases. As previously noted, *metJ* is only one of many simultaneous modes of methionine biosynthesis regulation. Given feedback-resistance at the entrance to the pathway, one could envision the *metJ* deletion strain performing slightly worse because of increased expression of downstream genes with minimal flux entering the pathway. Given that the RARE ∆*metJ* host performed better than the PTS^−^ glu^+^ RAREʹ ∆*metJ* host, we decided to continue with RARE ∆*metJ* for remaining experiments.

To further understand the limitations in methionine biosynthesis, we next supplemented the RARE ∆*metJ* host harboring the pathway with one of three amino acids: l-methionine, l-cysteine, and l-aspartate. Because cysteine and aspartate are precursors to methionine (Fig. 4a), our goal was to investigate whether reaction steps downstream of their biosynthesis were problematic. In this case, we added 10 mM of each amino acid at the time of induction (0 h) to see whether the time of supplementation would affect pathway kinetics. In this experiment, we also included the RARE host (with *metJ* intact) expressing the pathway in order to better understand the potential effect of *metJ* deletion. Performance of the RARE and RARE ∆*metJ* hosts supplemented with methionine was similar, with roughly 280 mg/L vanillate produced in just 24 h (Fig. 4c). Little additional vanillate formed after the first 24 h, suggesting that methionine supplementation at induction might be more effective than supplementation at 24 h. The decrease in the rate of vanillate formation suggested that all of the methionine added initially may have been depleted within 24 h. Addition of l-cysteine or l-aspartate did not improve vanillate titers, which supported the notion of next focusing on the reactions catalyzed by MetA and CysE. Interestingly, biomass formation occurred more rapidly than usual during l-aspartate supplementation, suggesting that uptake of this amino acid occurred readily but was diverted to other cellular purposes besides methionine biosynthesis (Additional file 1: Figure S2). On the other hand, addition of 10 mM l-cysteine significantly decreased titers and biomass formation. Cysteine has been shown to inhibit the growth of several *E. coli* strains by inhibiting threonine deaminase and therefore isoleucine synthesis [36].

We next explored whether expression of the metA* and cysE* genes would improve conversion of vanillate to protocatechuate without the addition of l-methionine to the culture medium (Fig. 4d). In the cases in which metA* and cysE* were overexpressed, average vanillate titer was observed to exceed average protocatechuate titer at all time points sampled. Expression of metA*−cysE* increased final vanillate titers by 33 % (from 205 ± 16 to 272 ± 35 mg/L). To gain insight into potential upper bounds of vanillate titer obtainable by increasing SAM availability, we supplemented cultures expressing metA*−cysE* with 10 mM methionine at both induction and at 24 h after induction (Additional file 1: Figure S3). In this case, vanillate titers continued to exceed protocatechuate titers at all time points sampled. Addition of methionine at induction led to 341 ± 25 mg/L of vanillate produced in the first 24 h. This suggested that further increased SAM availability within the first 24 h could still improve vanillate production. Although the addition of an extra 10 mM methionine (20 mM methionine total) at 24 h after induction resulted in a final vanillate titer of 419 ± 58 mg/L at 72 h after induction, protocatechuate titers remained above 100 mg/L. Thus, a strategy of additional exogenous methionine supplementation is insufficient to address the limitation of protocatechuate conversion, particularly once cultures have achieved late exponential or stationary phase. Given continued observations of a decreasing rate of vanillate formation after 24 h, we also investigated potential loss of the ampicillin-resistant plasmid harboring OMT (pET-AsbF-OMT). Based on the similar number of colonies appearing on plates taken at the same time points (Additional file 1: Figure S4), we could rule out plasmid loss as an explanation for the limited conversion observed.

### Orthogonal strategy of deregulating SAM regeneration through overexpression of *mtn* and *luxS* also achieves improvement in vanillate titers

**Fig. 5:**
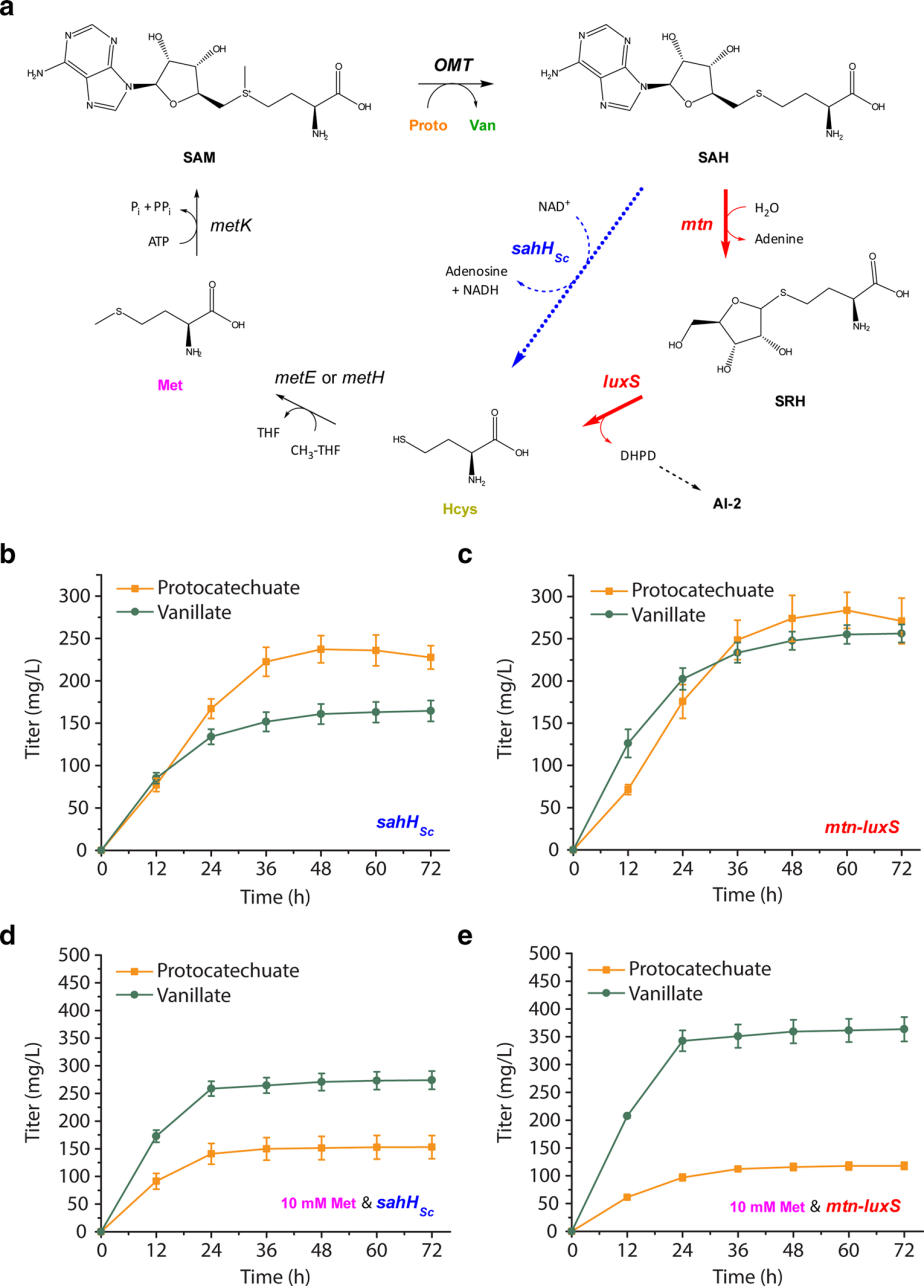
Orthogonal strategy of increasing *S*-adenosylhomocysteine (SAH) recycling through overexpression of *mtn* and *luxS* also improves vanillate titer. **a** The activated methyl cycle in *E. coli* (in *black* and *red*), along with an alternative SAH recycling route featuring a heterologous SAH hydrolase (*sahH*_*Sc*_, in *blue*). Native genes targeted for overexpression (*mtn* and *luxS*) are shown in *red*. **b** Effect of expressing *sahH*_*Sc*_ with aroG*,*asbF*_*Bt*_, *OMT*_*Hs*_ *, ppsA,* and *tktA* in the RARE ∆*metJ* host on protocatechuate and vanillate titers. **c** Effect of overexpressing *mtn* and *luxS* with aroG*,*asbF*_*Bt*_, *OMT*_*Hs*_ *, ppsA,* and *tktA* in the RARE ∆*metJ* host on protocatechuate and vanillate titers. **d** Effect of 10 mM methionine supplementation at induction on protocatechuate and vanillate titers using strains expressing *sahH*_*Sc*_. **e** Effect of 10 mM methionine supplementation at induction on protocatechuate and vanillate titers using strains overexpressing *mtn* and *luxS*

To our knowledge, no previous study has sought to increase SAM availability by targeting genes downstream of SAM utilization, in what is known as the activated methyl cycle. *S*-adenosyl-l-homocysteine (SAH) is a co-product of the reaction along with vanillate, and SAH is a potent inhibitor of SAM-dependent methyltransferases [37, 38] (Fig. 5a). Homocysteine supplementation results presented earlier suggested that recycling of SAH back to homocysteine does not occur at a rate sufficient to maintain SAM availability. One of the two reactions required to recycle SAH is catalyzed by Mtn and converts SAH to *S*-ribosyl-l-homocysteine (SRH). The subsequent conversion of SRH to homocysteine is catalyzed by LuxS and coupled to the production of autoinducer AI-2, which is a quorum sensing molecule [39, 40]. In *E. coli* and other bacteria, AI-2 has been implicated as an inducer of biofilm formation [41, 42] and as an attractant for chemotaxis [43]. Because recycling of SAH is linked to the formation of AI-2, SAM regeneration is expected to be subject to immense regulation. A recent LC–MS study profiled the effect of deletions of *mtn* and *luxS* on intracellular concentrations of SAM, SAH, SRH, homocysteine, and methionine at different OD_600_ values for *E. coli* MG1655 [44]. However, overexpression of these genes and the possible effect on intracellular concentrations of the same metabolites was not tested. We hypothesized that overexpression of *mtn* and *luxS* may increase SAM availability and therefore improve vanillate titers.

In eukaryotes, archaea, and non-LuxS-containing bacteria, SAM regeneration occurs differently, with conversion of SAH directly to homocysteine and adenosine catalyzed by SAH hydrolase (SAHase) [40]. We also hypothesized that overexpression of a heterologous SAHase might provide an alternative way to improve protocatechuate conversion with minimal interference to native regulation (Fig. 5a). To explore this concept, we tested expression of a SAHase from *S. cerevisiae* (*sahH*_*Sc*_). In all of the remaining experiments, neither metA* nor cysE* were expressed.

The two strategies intended to improve SAM regeneration had differing results. Final vanillate titers were 164 ± 12 mg/L when *sahH*_*Sc*_ was expressed (Fig. 5b), which is lower than the 205 ± 16 mg/L previously observed using a relevant control plasmid expressing no extra genes (Fig. 4d). Few conclusions should be drawn about the general concept of using a SAH hydrolase from the worsened pathway performance observed given that only one variant was tested and activity was not confirmed. Expression of *sahH*_*Sc*_ may have introduced a metabolic imbalance given its dependence on NAD^+^, or it may have simply added an expression burden if the gene product were inactive. However, when *mtn* and *luxS* were overexpressed, final vanillate titers were 256 ± 11 mg/L (Fig. 5c). The 25 % increase in average vanillate titers over the no expression control suggests that overexpression of the two native genes was effective at improving regeneration. A significant difference was also observed upon supplementing cultures containing each strain with 10 mM methionine at induction. Under these conditions, final vanillate titers were 274 ± 17 mg/L when *sahH*_*Sc*_ was expressed (Fig. 5d) and 364 ± 22 mg/L when *mtn* and *luxS* were overexpressed (Fig. 5e). For comparison, when 10 mM methionine was added to the control strain that did not overexpress these genes, final vanillate titers (at 48 h) were 299 ± 31 mg/L (Fig. 4c). Thus, overexpression of *mtn* and *luxS* improved vanillate titers in both the absence and presence of methionine supplementation. Table 1 contains a summary of protocatechuate and vanillate titers from key experiments, along with their cumulative titers.

**Table 1:** Summary of titers from key experiments

Number	Figure	Host	Genes overexpressed	Methionine added (mM)	Protocatechuate (P) Titer (mg/L)	Protocatechuate (P) Titer (mM)	Vanillate (V) Titer (mg/L)	Vanillate (V) Titer (mM)	Cumulative P+V Titer (mM)
1	1B	PTS-glu + RARE’	aroG*, asbF, OMT	0	216	1.4	135	0.8	2.2
2	1B	PTS-glu + RARE’	aroG*, asbF, OMT, ppsA, tktA	0	308	2.0	101	0.6	2.6
3	3B	PTS-glu + RARE’	aroG*, asbF, OMT, ppsA, tktA	0	323	2.1	101	0.6	2.7
4	4D	RARE ∆*metJ*	aroG*, asbF, OMT, ppsA, tktA	0	262	1.7	202	1.2	2.9
**5**	**4D**	**RARE ∆** ***metJ***	aroG*, asbF, OMT, ppsA, tktA, metA, cysE	**0**	**185**	**1.2**	**269**	**1.6**	**2.8**
6	5B	RARE ∆*metJ*	aroG*, asbF, OMT, ppsA, tktA, sahH	0	231	1.5	168	1.0	2.5
**7**	**5C**	**RARE ∆** ***metJ***	aroG*, asbF, OMT, ppsA, tktA, mtn, luxS	**0**	**277**	**1.8**	**252**	**1.5**	**3.3**
8	3B	PTS-glu + RARE’	aroG*, asbF, OMT, ppsA, tktA	10	246	1.6	168	1.0	2.6
9*	4C	RARE ∆*metJ*	aroG*, asbF, OMT, ppsA, tktA	10	200	1.3	303	1.8	3.1
10	5D	RARE ∆*metJ*	aroG*, asbF, OMT, ppsA, tktA, sahH	10	154	1.0	269	1.6	2.6
**11**	**5E**	**RARE ∆** ***metJ***	aroG*, asbF, OMT, ppsA, tktA, mtn, luxS	**10**	**123**	**0.8**	**370**	**2.2**	**3.0**
**12**	**S3**	**RARE ∆** ***metJ***	aroG*, asbF, OMT, ppsA, tktA, metA, cysE	**20**	**139**	**0.9**	**420**	**2.5**	**3.4**

Rows in bold formatting are highlighted in “Discussion” section

* Titers shown for experiment 9 correspond to samples obtained 48 h after induction, which was the final sampling time. All other titers correspond to samples obtained 72 h after induction

## Discussion

The results of this study present a detailed perspective on de novo vanillate biosynthesis in engineered *E. coli*. First, genetic perturbations were made upstream of the heterologous pathway in order to improve titers and better determine which reaction steps may be limiting. Upon identification of a limitation in the reaction catalyzed by OMT_*Hs*_, experiments were performed that provided several lines of evidence that SAM availability was the dominant cause of the limitation. Given that conversion of exogenously supplied homocysteine improved vanillate titers, attention was next focused on deregulating methionine biosynthesis by targeting the global regulator MetJ and the feedback-sensitive enzymes MetA and CysE. When deletion of *metJ* was coupled with expression of feedback-desensitized variants of metA* and cysE*, average de novo vanillate titers improved by 33 %. The orthogonal strategy of deregulating SAM regeneration by overexpressing *mtn* and *luxS* improved average de novo vanillate titers by 25 %. Overall, de novo vanillate titers improved roughly twofold over the course of experiments described in this manuscript.

In principle, the strategies of targeting biosynthesis or regeneration for deregulation provide different tradeoffs. Increasing SAM regeneration by increasing flux through the activated methyl cycle should be more efficient on a carbon basis than increasing flux through SAM biosynthesis because it depletes SAH, which is a toxic molecule and is known to inhibit *O*-methyltransferases, rather than potentially unbalancing central metabolic precursors. However, a potential disadvantage of increasing SAM regeneration is that the native *E. coli* pathway is coupled to biosynthesis of quorum sensing molecules that have widespread regulatory effects. The use of a yeast SAH hydrolase was motivated by the potential ability to decouple SAM regeneration and quorum sensing. The effectiveness of our implementation of these strategies can be determined from the titers shown in Table 1. Although overexpression of metA* and cysE* (Table 1, row #5) led to slightly higher vanillate titers than overexpression of *mtn* and *luxS* (Table 1, row #7), the latter strategy resulted in greater cumulative titers of protocatechuate and vanillate. In all cases, methionine supplementation led to a greater molar ratio of vanillate to protocatechuate (of note are rows #11–12). These results suggest that further efforts towards increasing SAM availability would be worthwhile for improving de novo production.

When compared with results from previously reported vanillin pathway optimization studies performed in yeast, our observations highlight the context dependence of engineered metabolic pathways. Two reports published after the initial demonstration of the engineered yeast vanillin pathway describe improvements obtained using flux balance analysis (FBA) model-guided optimization [8, 9]. In the first report, OptGene was used to identify target reactions that, if deleted, would increase vanillin production. Different deletion targets were suggested depending on the reference flux distribution in the minimization of metabolic adjustment (MOMA) biological objective function. In general, the predicted benefit from these modifications was increased availability of the co-factors ATP and NADPH required by Car_*Ni*_. Genes related to pyruvate metabolism, ammonium metabolism, the pentose phosphate pathway, and central carbon metabolism were identified. The only three modifications tested experimentally were deletion of one of the pyruvate decarboxylases (PDC1), deletion of the most active glutamate dehydrogenase (GDH1), and overexpression of GDH2 to ensure sufficient nitrogen uptake in the absence of GDH1. These modifications led to an overproducer strain that, when cultivated in a low dilution rate continuous fermentation, resulted in vanillin-β-d-glucoside titers of 500 mg/L [8]. As of that study, identification and overexpression of a potential rate-limiting enzyme in the pathway had not yet been described. In a second report, OMT_*Hs*_ and Car_*Ni*_ were overexpressed in the highest-producing strain obtained from the previous study [9] based on the observed accumulation of two heterologous intermediates (protocatechuate and protocatechualdehyde). Car_*Ni*_ overexpression did not lead to an increase in production, whereas OMT_*Hs*_ overexpression led to a 30 % increase in titer (now 380 mg/L vanillin-β-d-glucoside compared to a different baseline than referenced above). However, OMT_*Hs*_ overexpression in the parental strain from the first study did not alter titer. The authors concluded that this was likely because Car_*Ni*_ was limiting due to low availability of ATP and NADPH without the model-guided modifications made in the first study.

Given the many potential applications of methyltransferases in metabolic engineering [15, 16], the small number of reported examples in *E. coli* may reflect the limited host methylation capacity that we have encountered. In one report, a novel bacterial fatty acid methyltransferase was used to catalyze the formation of fatty acid methyl esters using free fatty acids and SAM [45]. The authors of that study note that SAM availability strongly regulated methyl ester production. By deleting the *metJ* gene mentioned earlier, and by overexpressing a gene encoding methionine adenosyltransferase from rat, the authors achieved an improvement in methyl ester production. However, the normalized titers of methyl esters in the supernatant achieved in their study increased from below 1 µM/OD to roughly 2.5 µM/OD. The corresponding amount of SAM required to achieve such conversion is orders of magnitude below what drives production of hundreds of milligrams per liter of vanillate.

Other reported examples of SAM-dependent methyltransferase reactions engineered in *E. coli* include several biotransformations to produce valuable methylated flavonoids, such as conversion of quercetin to rhamnetin [46], naringenin to 3-*O*-methylkaempferol [47], coumaric acid to 7-methoxyapigenin [48, 49], and coumaric acid to 7-*O*-methylaromadendrin [50]. Several of these methylated flavonoids are reported to have pharmacological properties. The largest titer reported in any of these examples was 111 mg/L of rhamnetin upon overexpression of *E. coli* SAM synthetase (*metK*) [46]. Results obtained in this study, such as those from methionine and homocysteine supplementation experiments, strongly suggest that conversion of methionine to SAM is not a primary limitation under native conditions of SAM biosynthesis. Rather, steps in upstream methionine biosynthesis and in downstream SAM regeneration appear to be limiting SAM utilization by heterologous methyltransferases, and these steps ought to receive greater initial focus for targeted deregulation. Strategies described in this work would likely further improve production of these valuable methylated flavonoids and other desired methylated compounds. In addition, based on the ease of detecting protocatechuate and vanillate, we propose that the vanillate pathway could serve as a convenient system in future studies that aim to understand and improve SAM biosynthesis and regeneration in *E. coli*.

## Conclusion

In this report, we have demonstrated that the de novo vanillin biosynthesis pathway engineered in *E. coli* suffers from a limitation in the conversion of protocatechuate to vanillate. Using several experimental approaches, we established that limited availability of the co-substrate SAM is the primary cause of the limitation. We then implemented two orthogonal strategies intended to increase SAM availability. One approach focused on deregulating SAM biosynthesis through *metJ* deletion and overexpression of feedback desensitized metA* and cysE*. The other approach aimed at deregulating SAM regeneration through overexpression of *mtn* and *luxS*. Both approaches led to increases in vanillate titer, and further increases were obtained through methionine supplementation. These results provide useful insights for improving production from pathways that involve methylation in *E. coli*, such as the vanillin pathway.

## Methods

### Strains and plasmids

*Escherichia coli* strains and plasmids used in this study are listed in Table 2. Molecular biology techniques were performed according to standard practices [51] unless otherwise stated. Molecular cloning and vector propagation were performed in DH5α. All host strains used for production experiments were derived from *E. coli* K-12 MG1655(DE3). In order to construct new host strains, two methods were used. The first was P1 transduction [52] using donor strains from the Keio collection [53] and P1 bacteriophage from ATCC (25404-B1). P1 transduction was used for all deletions of single genes. The second method was recombineering using the λ Red system [54]. Recombineering was used to delete the *yqhC*-*dkgA* operon and to upregulate *galP* expression by promoter substitution, as previously described [5]. Oligonucleotides were purchased from Sigma. Q5 High Fidelity DNA Polymerase (New England Biolabs, MA) was used for DNA amplification. In all cases of host strain modifications, pCP20 was used to cure the kanamycin resistance cassette [54].

**Table 2:** Strains and plasmids used in this study

Name	Relevant genotype	Source
*E. coli* strains		
DH5α	F^–^ Φ80*lac*ZΔM15 Δ(*lac*ZYA-*arg*F) U169 *rec*A1 *end*A1 *hsd*R17 (rK–, mK+) *pho*A *sup*E44 λ– *thi*-1 *gyr*A96 *rel*A1	Invitrogen
DH10B	F^−^ *mcr*A Δ(*mrr*-*hsd*RMS-*mcr*BC) Φ80*lac*ZΔM15 Δ*lacX74 recA1 endA1 araD139*Δ(*ara, leu*)7697 *galU* *galK* λ^−^ *rpsL nupG*	Invitrogen
MG1655	F^−^ λ^−^ *ilvG*- *rfb*-*50 rph*-*1*	ATCC 700926
MG1655(DE3)	F^−^ λ^−^ *ilvG*- *rfb*-*50 rph*-*1* (DE3)	Ref. [55]
RARE	MG1655(DE3) *∆dkgB ∆yeaE ∆(yqhC*-*dkgA) ∆yahK ∆yjgB*	Ref. [5]
RARE *∆metJ*	MG1655(DE3) *∆dkgB ∆yeaE ∆(yqhC*-*dkgA) ∆yahK ∆yjgB* *∆metJ*	This study
PTS^−^ glu^+^	MG1655(DE3) *∆ptsHIcrr* P_glk_::P_con*_ galP^q^	This study, but based on Ref. [56]
PTS^−^ glu^+^ RARE’	MG1655(DE3) *∆ptsHIcrr* P_glk_::P_con*_ galP^q^ *∆(yqhC*-*dkgA) ∆yahK ∆yjgB*	This study
PTS^−^ glu^+^ RARE’ *∆metJ*	MG1655(DE3) *∆ptsHIcrr* P_glk_::P_con*_ galP^q^ *∆(yqhC*-*dkgA) ∆yahK ∆yjgB ∆metJ*	This study
*E. coli* plasmids		
pCP20	λ cI857 (ts), λ *pr* Rep*ts*, Amp^R^, Cm^R^, λ p_r_ FLP	CGSC 7629
pKD13	*oriRγ*, Amp^R^, *kan*	CGSC 7633
pKD46	*oriR101, repA101* ^*ts*^, Amp^R^, *araC, araBp*-*λ*_*γ*_-*λ*_*β*_-*λexo*	CGSC 7739
pETDuet-1	Amp^R^, *lacI*, T7*lac*	Novagen
pACYCDuet-1	Cm^R^, *lacI*, T7*lac*	Novagen
pCOLADuet-1	Kan^R^, *lacI*, T7*lac*	Novagen
pCDFDuet-1	Str^R^, *lacI*, T7*lac*	Novagen
pACYC-*car*-*sfp*	pACYCDuet-1 harboring *car*_*opt*_ (carboxylic acid reductase from *Nocardia iowensis*, codon-optimized for expression in *E. coli*) and *sfp*_*opt*_ (phosphopantetheinyl transferase from *Bacillus subtilis*, codon optimized for expression in *E. coli*)	Ref. [5]
pET-*OMT*-*asbF*	pETDuet-1 harboring *Hs*-*S*-*COMT*_*opt*_ (catechol *O*-methyltransferase from *Homo sapiens*, codon-optimized for expression in *E. coli*) and *asbF*_*opt*_ (dehydroshikimate dehydratase from *Bacillus thuringiensis*, codon optimized for expression in *E. coli*)	Ref. [5]
pS4	Plasmid containing the shikimate module, version 4, kindly provided by the Keasling Lab at UC Berkeley. (Source of aroG*-*ppsA*-*tktA* artificial operon)	Ref. [57]
pACYC-aroG*	pACYCDuet-1 harboring the feedback-resistant aroG* from *E. coli*	This study
pACYC-aroG*-*ppsA*-*tktA*	pACYCDuet-1 harboring three *E. coli* genes in an artificial operon: aroG*, *ppsA*, and *tktA*	This study
pCOLA-*metK*	pCOLADuet-1 harboring the *E. coli metK* gene	This study
pET-*OMT*	pETDuet-1 harboring *Hs*-*S*-*COMT*_*opt*_	This study
pCOLA-metA*	pCOLADuet-1 harboring a feedback-desensitized version of *E. coli* *metA* (27_Arg- > Cys, 296_Ile- > Ser, and 298_Pro- > Leu, “metA*”)	This study
pCOLA-cysE*	pCOLADuet-1 harboring a feedback-desensitized version of *E. coli cysE* (95_Val- > Arg, and 96_Asp- > Pro, “cysE*”)	This study
pCOLA-metA*-cysE*	pCOLADuet-1 harboring an artificial operon consisting of the metA* and cysE* genes	This study
pCDF-*car*-*sfp*	pCDFDuet-1 harboring an artificial operon containing *car*_*opt*_ and *sfp*_*opt*_	This study
pCOLA-*mtn*	pCOLADuet-1 harboring the *E. coli mtn* gene	This study
pCOLA-*mtn*-*luxS*	pCOLADuet-1 harboring an artificial operon consisting of the *E. coli mtn* and *luxS* genes	This study
pCOLA-*sahH*	pCOLADuet-1 harboring *sahH*_*Sc*_ (*S*-adenosylhomocysteine hydrolase from *S. cerevisiae*, codon-optimized for expression in *E. coli*)	This study

The aroG*, *ppsA*, and *tktA* genes were kindly provided by Professor Jay D. Keasling at the University of California, Berkeley (USA). The genes encoding metA* and cysE* were synthesized as gBlocks (IDT, CA) and their sequences are included in Table 3. The gene encoding *sahH* from *S. cerevisiae* (UniProt #P39954) was codon-optimized for expression in *E. coli* and also synthesized as a gBlock (Table 3). The *E. coli metK*, *mtn*, and *luxS* genes were amplified from MG1655(DE3) genomic DNA using PCR amplication and the oligonucleotides shown in Table 4. All genes of interest were cloned into the Duet vector system (Novagen, WI) using restriction digest-based cloning. Restriction enzymes used to clone each gene are either shown in Table 3 (if the gene was synthesized) or in Table 4 (if the gene was amplified). Restriction enzymes and T4 DNA ligase were purchased from New England Biolabs. Propagated constructs were purified using a QIAprep Miniprep Kit (Qiagen, CA) and agarose gel fragments were purified using a Zymoclean Gel DNA Recovery Kit (Zymo Research, CA). All constructs were confirmed to be correct by nucleotide sequencing (Genewiz, NJ).

**Table 3:** Synthesized gene sequences used in this study

Gene (and restriction enzymes)	DNA sequence. Restriction enzyme sites are underlined and start/stop codons are in bold
metA* (NdeI/AatII)	AAAAAACAT **ATG**CCGATTCGTGTGCCGGACGAGCTACCCGCCGTCAATTTCTTGCGTGAAGAAAACGTCTTTGTGATGACAACTTCTTGTGCGTCTGGTCAGGAAATTCGTCCACTTAAGGTTCTGATCCTTAACCTGATGCCGAAGAAGATTGAAACTGAAAATCAGTTTCTGCGCCTGCTTTCAAACTCACCTTTGCAGGTCGATATTCAGCTGTTGCGCATCGATTCCCGTGAATCGCGCAACACGCCCGCAGAGCATCTGAACAACTTCTACTGTAACTTTGAAGATATTCAGGATCAGAACTTTGACGGTTTGATTGTAACTGGTGCGCCGCTGGGCCTGGTGGAGTTTAATGATGTCGCTTACTGGCCGCAGATCAAACAGGTGCTGGAGTGGTCGAAAGATCACGTCACCTCGACGCTGTTTGTCTGCTGGGCGGTACAGGCCGCGCTCAATATCCTCTACGGCATTCCTAAGCAAACTCGCACCGAAAAACTCTCTGGCGTTTACGAGCATCATATTCTCCATCCTCATGCGCTTCTGACGCGTGGCTTTGATGATTCATTCCTGGCACCGCATTCGCGCTATGCTGACTTTCCGGCAGCGTTGATTCGTGATTACACCGATCTGGAAATTCTGGCAGAGACGGAAGAAGGGGATGCATATCTGTTTGCCAGTAAAGATAAGCGCATTGCCTTTGTGACGGGCCATCCCGAATATGATGCGCAAACGCTGGCGCAGGAATTTTTCCGCGATGTGGAAGCCGGACTAGACCCGGATGTACCGTATAACTATTTCCCGCACAATGATCCGCAAAATACACCGCGAGCGAGCTGGCGTAGTCACGGTAATTTACTGTTTACCAACTGGCTCAACTATTACGTCTACCAGAGCACGCTATACGATCTACGGCACATGAATCCAACGCTGGAT**TAA** GACGTCAAAAAA
cysE* (AatII/XhoI)	AAAAAAGACGTCTAATAAAAGGAGATATACC**ATG**TCGTGTGAAGAACTGGAAATTGTCTGGAACAATATTAAAGCCGAAGCCAGAACGCTGGCGGACTGTGAGCCAATGCTGGCCAGTTTTTACCACGCGACGCTACTCAAGCACGAAAACCTTGGCAGTGCACTGAGCTACATGCTGGCGAACAAGCTGTCATCGCCAATTATGCCTGCTATTGCTATCCGTGAAGTGGTGGAAGAAGCCTACGCCGCTGACCCGGAAATGATCGCCTCTGCGGCCTGTGATATTCAGGCGGTGCGTACCCGCGACCCGGCAAGACCCAAATACTCAACCCCGTTGTTATACCTGAAGGGTTTTCATGCCTTGCAGGCCTATCGCATCGGTCACTGGTTGTGGAATCAGGGGCGTCGCGCACTGGCAATCTTTCTGCAAAACCAGGTTTCTGTGACGTTCCAGGTCGATATTCACCCGGCAGCAAAAATTGGTCGCGGTATCATGCTTGACCACGCGACAGGCATCGTCGTTGGTGAAACGGCGGTGATTGAAAACGACGTATCGATTCTGCAATCTGTGACGCTTGGCGGTACGGGTAAATCTGGTGGTGACCGTCACCCGAAAATTCGTGAAGGTGTGATGATTGGCGCGGGCGCGAAAATCCTCGGCAATATTGAAGTTGGGCGCGGCGCGAAGATTGGCGCAGGTTCCGTGGTGCTGCAACCGGTGCCGCCGCATACCACCGCCGCTGGCGTTCCGGCTCGTATTGTCGGTAAACCAGACAGCGATAAGCCATCAATGGATATGGACCAGCATTTCAACGGTATTAACCATACATTTGAGTATGGGGATGGGATC**TAA** CTCGAGAAAAAA
*sahH*_*Sc*_ (NcoI/NotI)	ATATATCC **ATG**GGCGCCCCTGCTCAGAATTATAAAATTGCAGATATCTCACTGGCAGCGTTCGGTCGTAAGGAAATTGAATTGGCAGAGCACGAAATGCCAGGCCTGATGGCAATTCGTAAAGCCTATGGCGACGTTCAACCGCTGAAGGGCGCACGTATCGCGGGTTGCTTACACATGACGATCCAAACCGCCGTGCTGATTGAGACATTAGTCGCTTTGGGAGCCGAGGTTACGTGGTCGTCATGCAATATCTACTCGACGCAAGATCATGCGGCAGCGGCAATCGCTGCAAGCGGCGTGCCTGTGTTTGCTTGGAAAGGTGAAACCGAAGAGGAATACCTGTGGTGCATTGAACAGCAGCTGTTTGCATTCAAGGATAACAAAAAATTGAACCTGATTTTGGACGACGGCGGGGATTTAACAACCTTAGTTCACGAGAAACATCCTGAAATGCTGGAGGATTGTTTTGGCCTTTCCGAAGAAACCACCACCGGTGTCCACCACCTCTACCGCATGGTTAAAGAGGGTAAACTTAAAGTCCCGGCTATCAATGTGAACGACTCAGTAACCAAATCCAAATTTGATAACTTATATGGTTGCCGTGAAAGCCTGGTTGACGGGATTAAGCGCGCCACGGATGTTATGCTTGCCGGAAAGGTGGCTGTTGTGGCCGGTTACGGTGACGTGGGTAAAGGATGCGCAGCCGCGTTACGTGGCATGGGTGCACGCGTGTTAGTTACGGAAATTGATCCTATCAATGCCCTGCAGGCCGCGATGGAAGGCTATCAAGTGGTAACAATGGAAGATGCCTCACATATCGGTCAGGTGTTCGTTACGACCACGGGTTGTCGTGATATCATCAACGGGGAGCATTTCATTAACATGCCGGAAGATGCAATTGTTTGTAACATCGGGCATTTCGATATTGAGATCGACGTGGCGTGGCTCAAAGCCAATGCCAAAGAGTGTATTAACATTAAACCGCAGGTAGATCGTTATTTGCTGAGCTCTGGCCGTCATGTCATCCTCCTGGCAAATGGACGCCTTGTGAATCTGGGTTGTGCGACGGGTCACTCGTCCTTCGTCATGAGCTGCTCTTTTTCCAACCAGGTTCTGGCACAAATCGCCCTGTTTAAATCTAATGACAAATCTTTTCGCGAAAAACACATCGAGTTCCAGAAGACCGGTCCCTTCGAAGTCGGGGTTCATGTGTTGCCTAAAATCTTAGATGAGGCAGTCGCTAAATTTCATCTTGGCAACCTGGGTGTCCGTCTGACCAAACTGAGTAAAGTCCAGAGCGAGTACCTGGGTATTCCGGAGGAAGGACCTTTCAAAGCGGATCATTATCGCTAT**TAA** GCGGCCGCAAATTT

**Table 4:** Oligonucleotides used in this study

Name	Sequence (5′– >3′)
MetJ-verify-f	TCTTTAGCAATCACCACG
MetJ-verify-r	GGAATATTCTTGCCGTAAC
PtsHICrr-verify-f	GAAAGGCGCAATCCAA
PtsHICrr-verify-r	CGATTTGACTGCCAGAAT
AroG*-f (BglII)	AAAAAAAGATCTGATGAATTATCAGAACGACGATTTAC
AroG*-r (AvrII)	AAAAAACCTAGGCCTCCTTTAGATCCTTACCC
AroG*-PpsA-TktA-f (BglII)	AAAAAAAGATCTGATGAATTATCAGAACGACGATTTAC
AroG*-PpsA-TktA-r (AvrII)	AAAAAACCTAGGTTACAGCAGTTCTTTTGCTTTC
MetK-f (NdeI)	AAAAAACATATGGCAAAACACCTTTTTAC
MetK-r (AvrII)	AAAAAACCTAGGTTACTTCAGACCGGCAG
Mtn-f (NcoI)	AAAAAACCATGGGCAAAATCGGCATCATTGG
Mtn-r (NotI)	AAAAAAGCGGCCGCTTAGCCATGTGCAAGTTTCT
LuxS-f (NotI)	AAAAAAGCGGCCGCTAATAAAGGAGATATACCATGCCGTTGTTAGATAGCTT
LuxS-r (AflII)	AAAAAACTTAAGCTAGATGTGCAGTTCCTGC

### Chemicals

The following compounds were purchased from Sigma: vanillic acid, protocatechuic acid (otherwise known as 3,4-dihydroxybenzoic acid), l-methionine, l-homocysteine, l-cysteine, and l-aspartate. Isopropyl β-d-1-thiogalactopyranoside (IPTG) was purchased from Denville Scientific. Ampicillin sodium salt, chloramphenicol, streptomycin sulfate, and kanamycin sulfate were purchased from Affymetrix.

### Culture conditions

A 1X M9 salt medium (Sigma) containing 6.78 g/L Na_2_HPO_4_∙7H_2_O, 3 g/L KH_2_PO_4_, 1 g/L NH_4_CI, and 0.5 g/L NaCl, supplemented with 2 mM MgSO_4_, 0.1 mM CaCl_2_, glucose, trace elements, and antibiotics was used as the culture medium for experiments in this study. The trace element solution (100×) used contained 5 g/L EDTA, 0.83 g/L FeCl_3_∙6H_2_O, 84 mg/L ZnCl_2_, 10 mg/L CoCl_2_∙6H_2_O, 13 mg/L CuCI_2_∙2H_2_O, 1.6 mg/L MnCl_2_∙2H_2_O and 10 mg/L H_3_BO_3_ dissolved in water. This was added to a concentration of 1× to supplement the M9-glucose medium. This medium will be henceforth referred to as “M9-glu-trace.” For all experiments except the experiment corresponding to Fig. 1b, the initial glucose concentration was 1.8 %. The initial glucose concentration was 1.2 % for the experiment corresponding to Fig. 1b.

All experiments were performed in 250 ml baffled PYREX shake flasks that contained 50 ml culture volumes. Overnight cultures were grown in 3 ml in 14 ml round-bottom tubes (Corning). Experimental cultures were initiated as follows: 1 % (v/v) inoculum volumes of overnight culture in LB medium were first transferred into overnight culture in M9-glu-trace medium, and then 1 % (v/v) inoculum volumes of overnight culture in M9-glu-trace were transferred into 50 mL M9-glu-trace medium, incubated at 30 °C, and agitated at 250 rpm. The OD_600_ was measured regularly during exponential growth using a DU800 UV/Vis spectrophotometer (Beckman Coulter). Cultures were induced with 0.5 mM IPTG at OD_600_ values between 0.8 and 1.0. Depending on the experiment, culture medium was supplemented with either 50 mg/L ampicillin, 17 mg/L chloramphenicol, 25 mg/L streptomycin, 25 mg/L kanamycin, or combinations of the previous antibiotics to provide selective pressure for plasmid maintenance. In some experiments (Figs. 4, 5; Additional file 1: Figures S2–S4), 50 mg/L carbenicillin was used in place of ampicillin in liquid cultures to provide more stringent pressure for plasmid maintenance. All experiments were performed in biological triplicate, and results are presented as averages with error bars representing one standard deviation.

For experiments in which *S*-adenosyl-l-methionine precursors were supplemented, flask cultures were set up as mentioned before but with culture volumes adjusted to achieve final concentrations as follows: 10 mM l-methionine, 2.5 mM l-homocysteine, 10 mM l-cysteine, or 10 mM l-aspartate. Stocks of supplemented metabolites were pH-neutralized and sterile filtered. In these experiments, control cultures received an equal volume of sterile deionized water instead of the metabolic precursors at the time of supplementation. Supplementation times varied from occurring at induction (0 h), occurring 24 h after induction, and occurring at both induction and 24 h after induction.

### Metabolite analysis

Culture samples were pelleted by centrifugation and aqueous supernatant was collected for HPLC analysis using an Agilent 1100 series instrument equipped with a diode array detector. Heterologous compounds produced in vanillin experiments were separated using a Zorbax Eclipse XDB-C18 column (Agilent) and detected using a wavelength of 280 nm. A gradient method used the following solvents: (A) 50 % acetonitrile+ 0.1 % trifluoroacetic acid (TFA); (B) water+ 0.1 % TFA. The gradient began with 5 % Solvent A and 95 % Solvent B. The setting at 20 min was 60 % Solvent A and 40 % Solvent B. The program restored the original ratio at 22 min and ended at 25 min. The flow rate was 1.0 ml/min and all vanillin pathway compounds of interest eluted within 15 min. Column temperature was maintained at 30 °C.

### SDS–PAGE analysis

To determine qualitative protein expression level of OMT in the absence of other pathway gene overexpression, *E. coli* MG1655(DE3) was transformed with empty pETDuet-1 or pET-OMT. Single colonies from plates of each transformation were grown overnight in 3 ml of LB with 50 mg/L ampicillin. Cells were passaged into second overnight cultures by inoculating 3 ml of M9+ 1.8 % glucose with 100 μL of the overnight LB cultures. Shake flask cultures containing 50 ml M9+ 1.8 % glucose were inoculated at 1 % inoculum from overnight M9 cultures and incubated with agitation at 30 °C and 250 rpm. Cultures were induced with 0.5 mM IPTG at OD_600_ values between 0.8 and 1.0. Twenty-four hours after induction, 5 ml of each culture were sampled and pelleted by centrifugation. Cell pellets were resuspended in 1 ml of 10 mM Tris–HCl at pH 8.0 and lysed using sonication. After lysis, samples were pelleted by centrifugation (16,000*g*, 4 °C, 20 min) and supernatant was collected as soluble lysate. The remaining pellet was resuspended in 10 mM Tris–HCl and deemed the insoluble fraction.

Total protein was quantified by the Bradford assay method [58] using Bio-Rad Protein Assay Dye Reagent (Cat #500-0006) and a bovine serum album (BSA) standard. A Bio-Rad 10 % Mini-PROTEAN TGX gel (Cat #456-1034) was run using the Mini-PROTEAN Tetra Cell electrophoresis apparatus. Bio-Rad Precision Plus Protein All Blue Standard (Cat #161-0373) and 10 μg of total protein for each sample was loaded on the gel. After running at 200 volts for 33 min, the gel was washed with deionized water before staining with Bio-Rad Bio-Safe Coomassie Stain (Cat #161-0786).

### *O*-methyltransferase in vitro specific activity

To determine the specific activity of the *O*-methyltransferase as a function of time in the context of the pathway, *E. coli* MG1655(DE3) was transformed with pET-OMT-AsbF and pACYC-AroG*-PpsA-TktA plasmids. Single colonies from plates of each transformation were grown overnight in 3 ml of LB with 50 mg/L ampicillin and 17 mg/L chloramphenicol. Cells were passaged into second overnight cultures by inoculating 3 ml of M9+ 1.8 % glucose+ antibiotics with 100 μL of the overnight LB cultures. Shake flask cultures containing 50 ml M9+ 1.8 % glucose+ antibiotics were inoculated at 1 % inoculum from overnight M9 cultures and incubated with agitation at 30 °C and 250 rpm. Cultures were induced with 0.5 mM IPTG at OD_600_ values between 0.8 and 1.0. At 12 h time point increments after induction, 5 ml of each culture were sampled and pelleted by centrifugation. Cell pellets were resuspended in 1 ml of 100 mM sodium phosphate buffer at pH 7.5 and lysed using sonication. After lysis, samples were pelleted by centrifugation (16,000*g*, 4 °C, 20 min) and supernatant containing OMT was collected.

The OMT activity assay contained the following final concentrations to a final volume of 600 µL per sample: 100 mM sodium phosphate buffer (pH 7.5), 2 mM protocatechuate, 2 mM *S*-adenosylmethionine, 5 mM MgCl_2_, and roughly 0.1 mg/mL BSA protein equivalent of lysate (60 µL). Lysate was always added last, and immediately upon addition, the assay solution was split into two 300 µL volumes (t_i_ and t_f_). The t_i_ volume was quenched with 1 % TFA, and the t_f_ was quenched in the same manner after 1 h. Both volumes for each sample were maintained at 30 °C. Potential debris from quenched volumes were pelleted by centrifugation (16,000*g*, 4 °C, 3 min) and supernatant was analyzed by HPLC. Concentrations of protocatechuate and vanillate were measured as described before. Specific OMT activity was calculated by dividing differences in vanillate concentration by total protein concentration in corresponding lysate. Specific activities were subsequently normalized to the activity at the first time point sampled (12 h). Lysate from a strain transformed with empty pET was used as a control in otherwise identical assays to ensure there was no background conversion of protocatechuate.

### Calculation of maximum theoretical pathway yield

Maximum theoretical yields presented for vanillin and vanillate were calculated using an in silico genome-scale metabolic model of *E. coli* (iJO1366) [59] and the constraint-based reconstruction and analysis (COBRA) toolbox version 2.0 [60, 61] within the framework of MATLAB 2013a. Heterologous vanillin pathway reactions were added to the model, and uptake rates for glucose and oxygen were set to −10 mmol/gDW-h and −1000 mmol/gDW-h, respectively [62].

## Additional file


10.1186/s12934-016-0459-x Supplementary material
