# Supplementary material for: Deregulation of S-adenosylmethionine biosynthesis and regeneration improves methylation in the E. coli de novo vanillin biosynthesis pathway
Source: Microb Cell Fact. 2016 Apr 11;15:61. doi: 10.1186/s12934-016-0459-x (PMC4828866; doi:10.1186/s12934-016-0459-x)
Supplement: Supplementary file 1 — 10.1186/s12934-016-0459-x Supplementary material [file 12934_2016_459_MOESM1_ESM.docx]

**Supplementary Material:** Deregulation of *S*-adenosylmethionine biosynthesis and regeneration improves methylation in *E. coli de novo* vanillin pathway

**Authors:** Aditya M. Kunjapur^†^, Jason C. Hyun, Kristala L. J. Prather

**Affiliations:**

Department of Chemical Engineering, Massachusetts Institute of Technology, Cambridge, MA 02139, USA

Synthetic Biology Engineering Research Center (SynBERC), Massachusetts Institute of Technology, Cambridge, MA 02139, USA

^†^ Present address: Department of Genetics, Harvard Medical School, Boston, MA 02115

**Figures:**


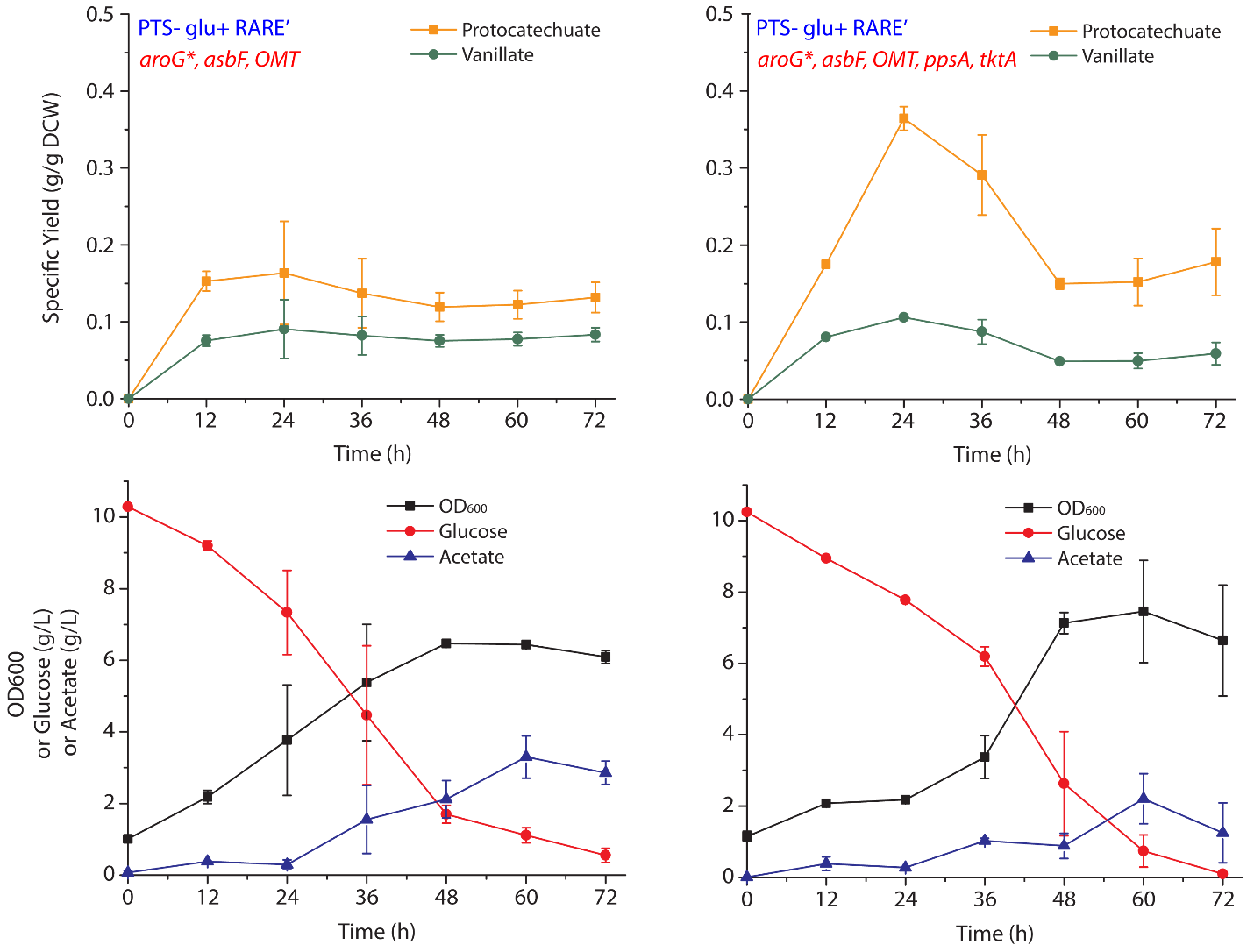


**Figure S1.** Effect of *ppsA* and *tktA* overexpression on specific yields of protocatechuate and vanillate, as well as the effect on culture density, glucose consumption, and acetate production. The increased protocatechuate specific yield from 24-36 h with *ppsA* and *tktA* overexpression indicates greater production of protocatechuate per cell rather than a greater number of cells.


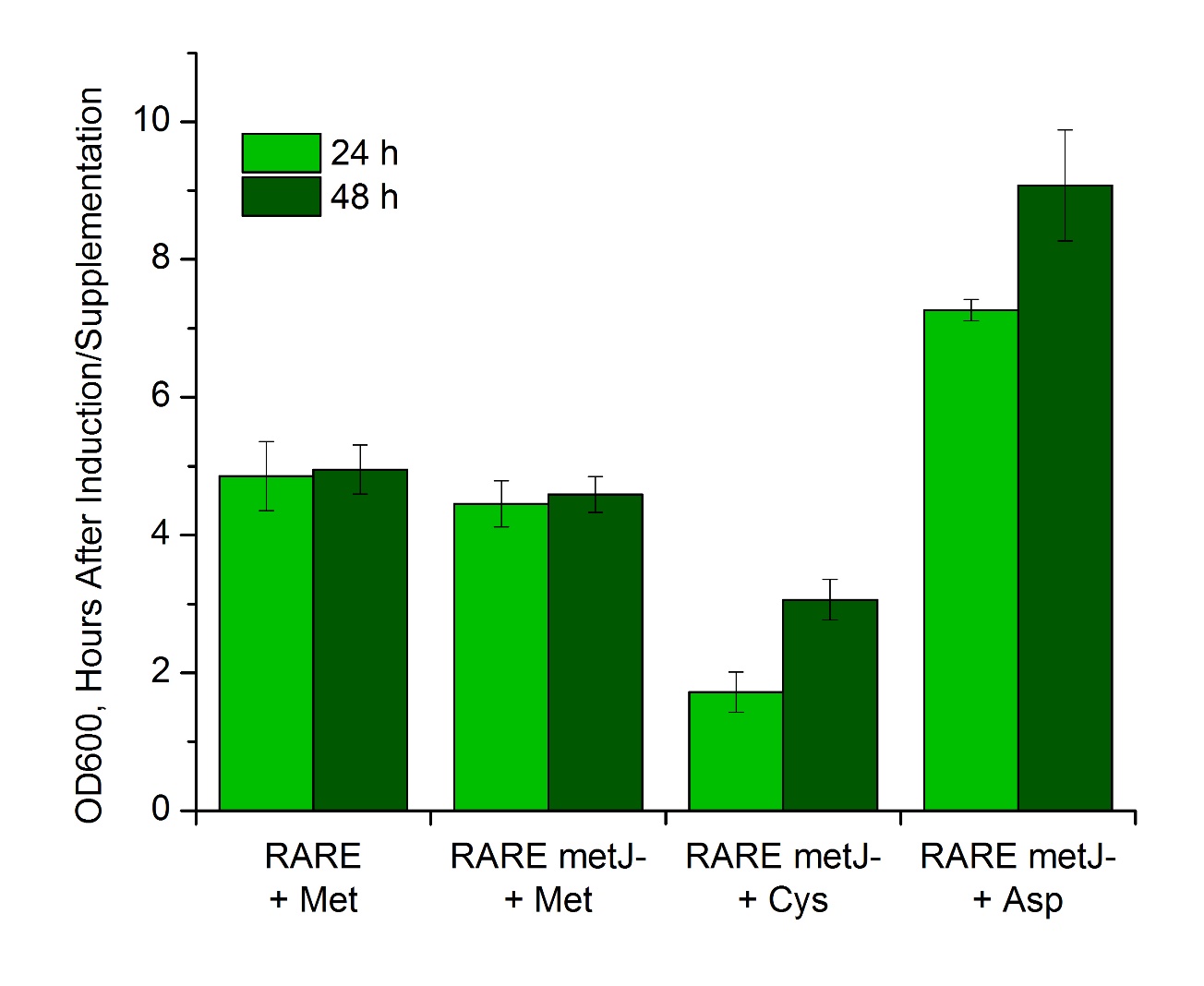


**Figure S2.** OD_600_ values for cultures supplemented with different amino acids. Cultures subjected to cysteine supplementation displayed significant impairment of biomass formation, whereas cultures subjected to aspartate supplementation displayed accelerated biomass formation and greater final cell density.

**
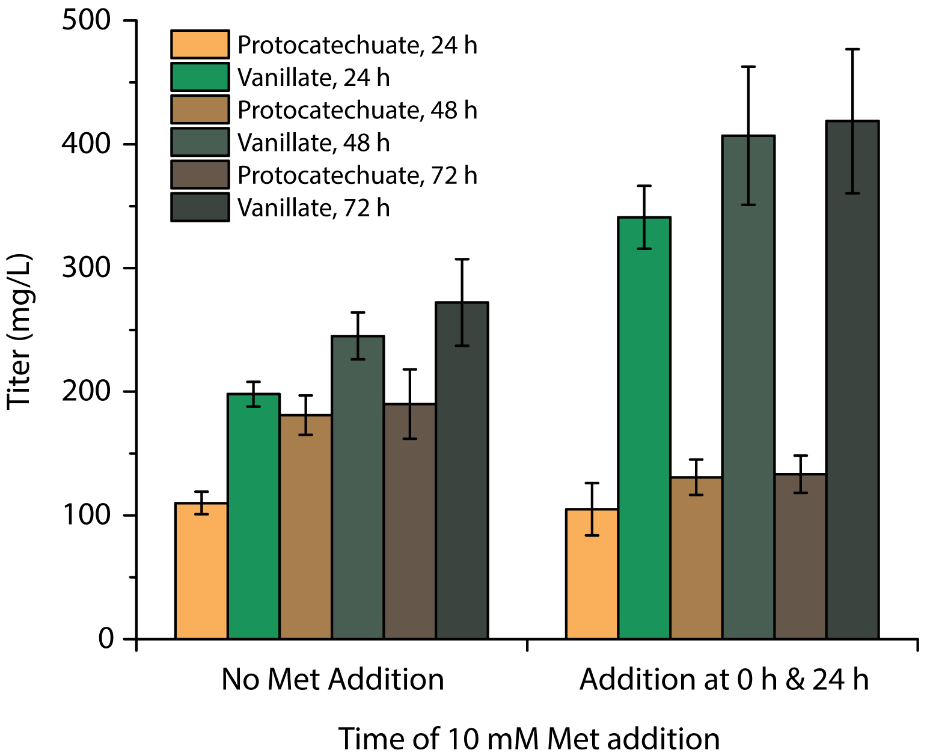
**

**Figure S3.** Effect of methionine supplementation on cultures containing cells expressing *metA** and *cysE**.


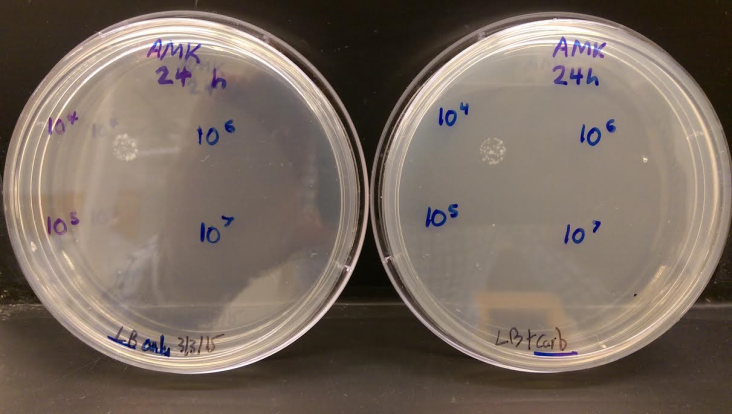


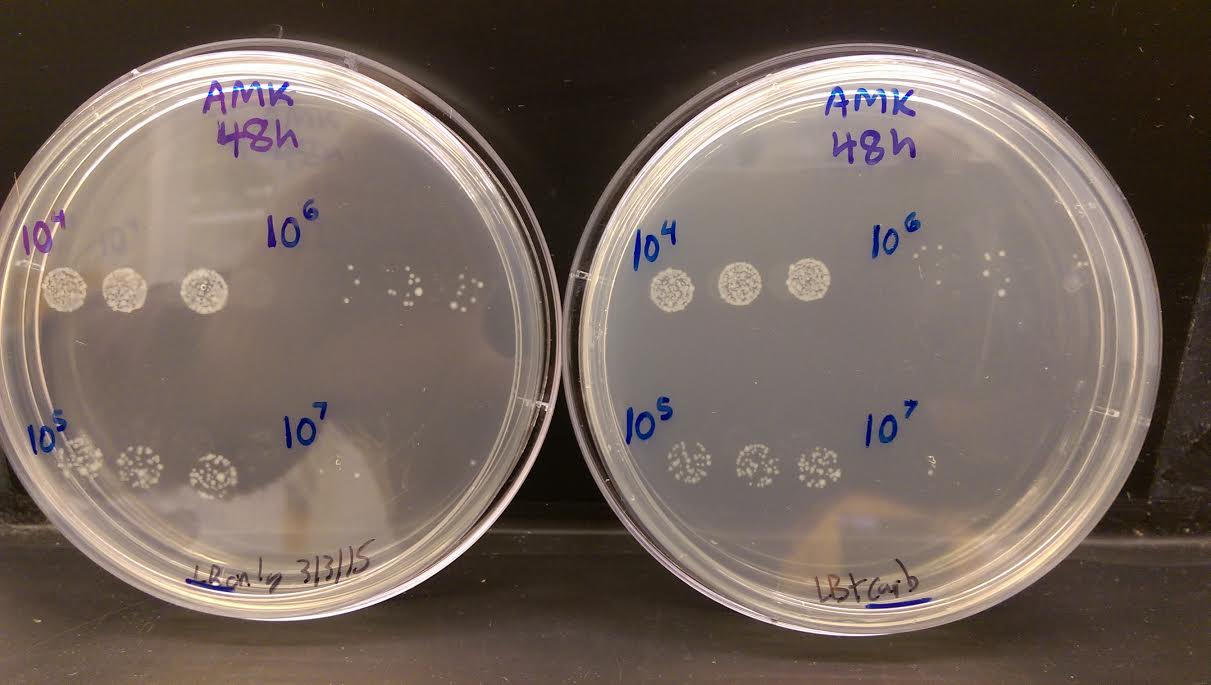


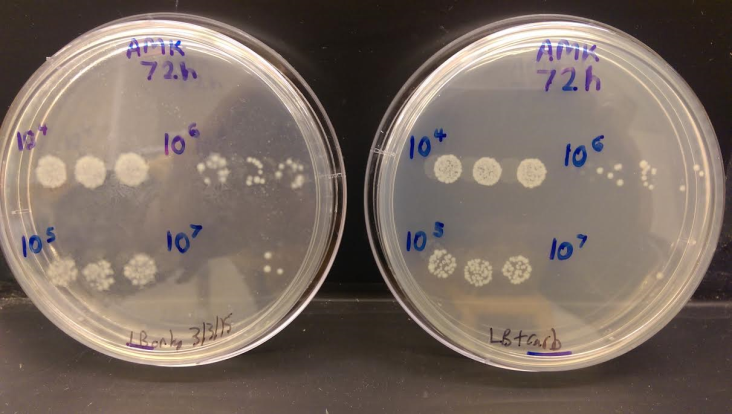


**Figure S4.** Images of plates testing for potential loss of ampicillin-resistant plasmid. Cells were sampled from cultures overexpressing *metA**­*-cysE** at 24, 48, and 72 hours. Each sample was serially diluted in sterile deionized water to the range of 10^4^-10^7^ fold and then immediately plated by pipetting 10 uL droplets on plates containing LB or LB and 100 mg/L carbenicillin. Carbenicillin was used instead of ampicillin for a more stringent selection. No significant plasmid loss was observed for samples taken at 24, 48, and 72 h.
